# Ecological patterns and spatial distribution of medicinal mollusks in a freshwater ecosystem

**DOI:** 10.1186/s13071-026-07273-9

**Published:** 2026-02-18

**Authors:** Junhua Liu, Lifan Xiao, Jianfeng Zhang, Beixin Wang, Ming Du, Jun Chen, Jinming Zhu, Liang Shi, Kun Yang

**Affiliations:** 1 Wuxi Binhu District Center for Disease Control and Prevention, Wuxi, 214063 China; 2 National Health Commission of Key Laboratory for Parasitic Disease Prevention and Control, Jiangsu Provincial Key Laboratory On Parasite and Vector Control Technology, Jiangsu Institute of Parasitic Diseases, Jiangsu, 117 Yangxiang, Meiyuan, Wuxi, 214064 China; 3 Laboratory of Aquatic Insects and Stream Ecology, Department of Entomology, Nanjing Agricultural University, Nanjing, 210095 China; 4 Fudan University School of Public Health, Key Laboratory of Public Health Safety, Ministry of Education,Fudan University Center for Tropical Disease Research, Fudan University, No. 220 Handan Road, Yangpu District, Shanghai, 200032 China

**Keywords:** Medicinal mollusks, Lake Taihu, Spatial aggregation, GIS analysis, Parasitic diseases, Mollusk monitoring

## Abstract

**Background:**

The Lake Taihu wetland in the Yangtze River Delta is a key freshwater ecosystem. Medicinal mollusks act as intermediate hosts for multiple parasitic diseases, yet their distribution patterns and public health implications along the northern shore of Lake Taihu remain insufficiently characterized.

**Methods:**

Field surveys were conducted between 2022 and 2023 at 48 sampling sites in Zhushan Bay, Meiliang Bay, and Gonghu Bay. A total of 1605 mollusk samples were collected using multiple sampling methods. Species were identified morphologically, and diversity indices were calculated. Spatial hotspots were analyzed using ArcGIS, and a mollusk-parasite network was constructed using Cytoscape. Inter-bay differences were assessed using statistical analyses.

**Results:**

Twenty mollusk species were identified, with Viviparidae showing the highest density and Planorbidae the greatest species richness. *Sinotaia aeruginosa* was the dominant species. *Radix swinhoei* and *R. plicatula* occupied key positions within the parasite transmission network. Zhushan Bay exhibited the lowest community evenness, and hotspot analysis identified spatially aggregated high-risk areas.

**Conclusions:**

Medicinal mollusks along the northern shore of Lake Taihu exhibit high diversity and density, with Zhushan Bay representing a critical area for parasite transmission. Targeted control strategies focusing on key host species and high-risk ecological nodes are recommended to support risk early-warning systems and reduce regional mollusk-borne parasitic disease risks.

**Graphical Abstract:**

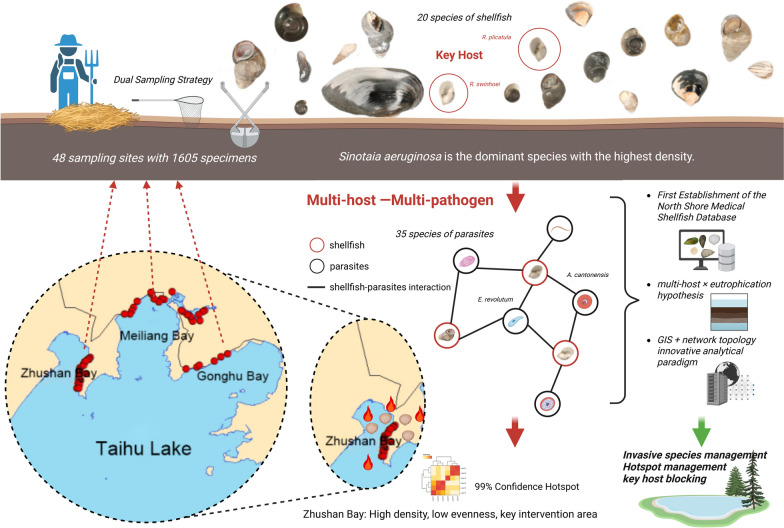

**Supplementary Information:**

The online version contains supplementary material available at 10.1186/s13071-026-07273-9.

## Background

Medicinal mollusks inhabiting freshwater or brackish environments are mainly gastropod species that act as obligate intermediate hosts for a wide range of parasites (e.g., trematodes, nematodes). These mollusks are indispensable components of the life cycles of many parasitic helminths and play a pivotal role in the transmission of parasitic diseases, particularly zoonotic infections, by facilitating parasite development and dissemination within aquatic ecosystems [1–3]. In China, 13 families, 29 genera, and 78 species of mollusks with documented or potential roles in parasite transmission have been identified. These mollusks are widely distributed across various aquatic environments, such as rivers, lakes, and rice fields, creating favorable conditions for the maintenance and spread of snail-borne parasitic diseases.

In addition to serving as intermediate hosts of parasites, certain gastropod species are directly used as medicinal materials, commonly referred to as “medicinal mollusks,” particularly in traditional Chinese medicine [4]. Historically, these species have been employed for detoxification, anti-inflammatory, and diuretic purposes, with common forms of application including decoctions for oral administration and powders and extracts for external use [5, 6]. Owing to their long-standing cultural foundation and potential pharmacological activities, medicinal mollusks remain widely accessible through folk practices and online markets [7]. However, systematic evaluations of their associated health risks remain limited. Existing concerns primarily involve species misidentification and substitution or adulteration, harvesting and processing practices, and contamination by microorganisms, parasites, heavy metals, persistent environmental pollutants, algal toxins, and pharmaceutical residues [8]. In this context, the present study investigates the potential health risks of medicinal mollusks across their sources, processing procedures, and modes of use, aiming to provide evidence-based guidance for safe utilization and regulatory oversight.

Emerging zoonotic protozoan diseases, such as babesiosis, indicate that pathogen transmission within aquatic ecosystems may involve multiple intermediate hosts, including freshwater snails, and is increasingly influenced by climatic and anthropogenic changes [3, 9]. Studies on economically important mollusk species further reveal cryptic protozoan transmission pathways and associated diagnostic challenges [10], while advances in molecular detection techniques have provided more sensitive tools for the early identification of parasitic pathogens [11]. Collectively, these findings underscore broader One Health implications, emphasizing that mollusk species with direct relevance to human health, including medicinal freshwater mollusks, warrant systematic evaluation for potential parasitic and protozoan risks within dynamic host-pathogen-environment networks.

The Taihu Lake wetland, located in the Yangtze River Delta, is the largest plain freshwater lake wetland in China, covering ~ 2250 km^2^ [12]. Its dense water networks and heterogeneous habitats provide favorable breeding and maintenance conditions for freshwater mollusks [13]. However, intensified human disturbance and increased human contact with water in recent years have substantially elevated public health risks associated with water- and snail-borne parasitic diseases [14]. Despite advances in freshwater mollusk research, systematic investigations of medicinal mollusks remain limited, particularly along the northern shore of Lake Taihu, where species diversity is high and utilization is widespread. Therefore, this region represents both a key resource hotspot and a priority area for parasitological surveillance. As intermediate hosts for multiple parasites, the spatial distribution and population structure of mollusks directly influence patterns of parasite transmission and disease risk [15]. Consequently, identifying dominant species, population clusters, and distribution hotspots is essential for effective surveillance and early warning systems [16]. Human activities have substantially altered the hydrological structure and functional connectivity of river networks across the Taihu Plain, reshaping water flow patterns and ecosystem processes that are closely linked to parasite life cycles and host availability [17]. In Lake Taihu, these cumulative impacts interact with pressures from agricultural runoff and aquaculture [18], leading to increased nutrient and pollutant inputs, altered water quality and microbial community composition, and changes in host ecology. Together, these processes connect eutrophication, algal blooms, and pathogen dynamics, thereby amplifying potential public health threats.

This study did not involve microscopic or molecular parasitological analyses, and parasite-host information was obtained from published literature. Accordingly, the work represents a baseline ecological and spatial assessment rather than a direct parasitological investigation. In light of the current state of research and the need for effective disease control, this study aims to establish the first comprehensive baseline database of medicinal mollusks along the northern shore of Lake Taihu through multi-site continuous monitoring conducted between 2022 and 2023. Methodologically, the study integrates D-net quantitative sampling with the straw curtain method to develop a rapid, highly sensitive mollusk-monitoring paradigm suitable for shallow lake shores. From a spatial epidemiological perspective, this study introduces the Getis-Ord Gi* hotspot analysis at a 500-m spatial resolution to medicinal mollusk research for the first time to our knowledge, allowing fine-scale identification and visualization of mollusk aggregation patterns relevant to parasite transmission. In addition, the study integrates mollusk host spectra, water quality indicators, and indices of human disturbance to propose a “host–pathogen-environment” triadic One-Health risk model, providing data support and methodological reference for the precise early warning, risk assessment, and targeted intervention of mollusk-mediated parasitic diseases.

## Methods

### Study area

**Fig. 1:**
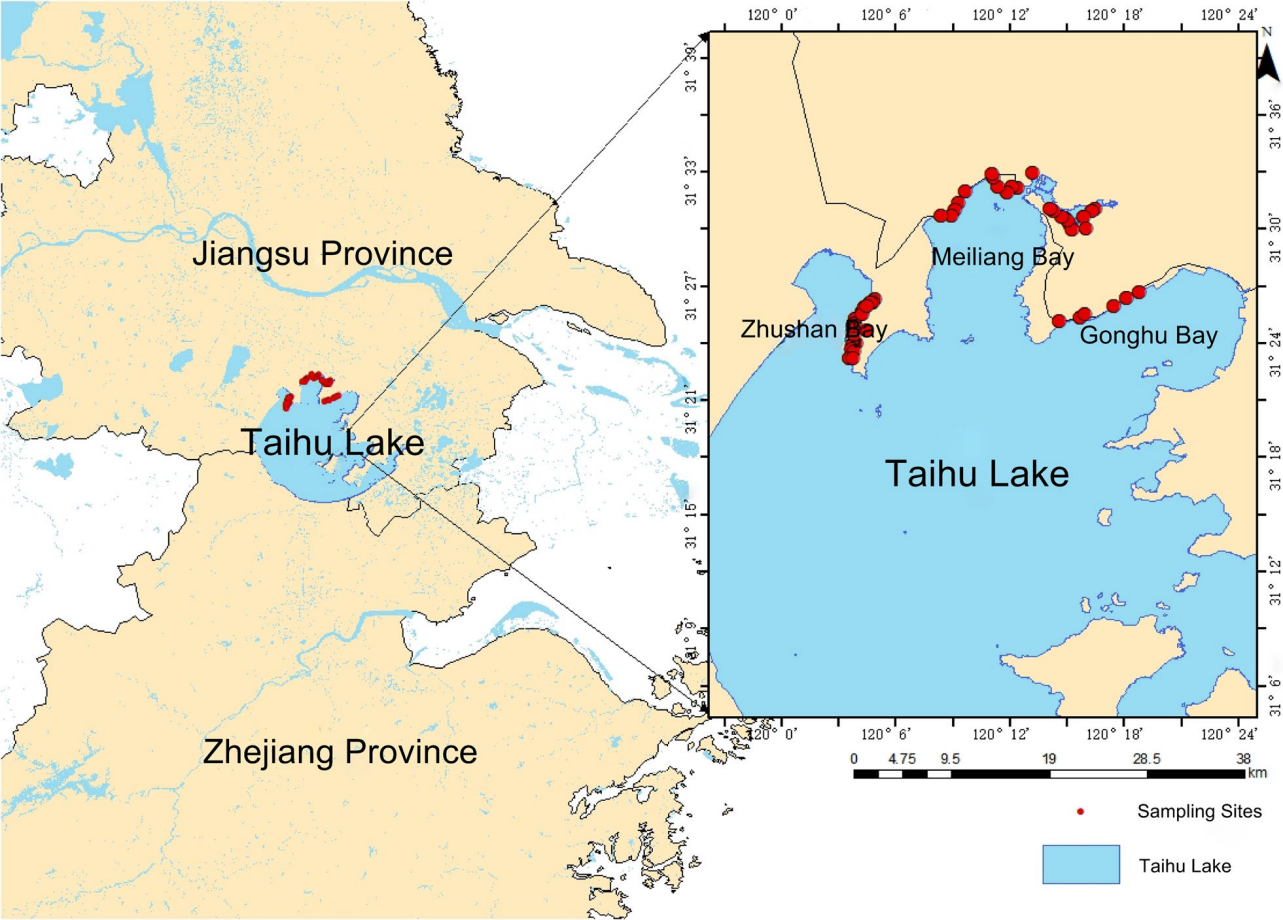
Distribution of medicinal mollusk sampling sites in the Lake Taihu wetland

The northern shore of Lake Taihu in Jiangsu Province was selected as the study area. A total of 48 sampling sites were established across Zhushan Bay, Meiliang Bay, and Gonghu Bay in the northern region of the lake. Sampling locations encompassed multiple habitat types relevant to freshwater mollusk distribution, including lakeshore rocky zones, ponds, rivers, and streams (Table 1). Field sampling was conducted during two survey periods, in October 2022 and July to August 2023. Sampling sites were selected based on the spatial configuration of the water system to adequately capture mollusk occurrence across connected aquatic habitats. Mollusk specimens were collected from all relevant microhabitats at each site. The spatial distribution of sampling points was as follows: 18 sites in Zhushan Bay, 24 sites in Meiliang Bay, and 6 sites in Gonghu Bay (Fig. 1, Table S1).

**Table 1:** Habitats of sampling sites in the wetlands surrounding Lake Taihu

Wetland monitoring sites around Taihu Lake (River name)	Number of sampling points	Habitat
Zhushan Bay	19	Lakeshore rocky zones, rivers, ponds, streams, and parks
Meiliang Bay	24	Ditches, lakeshore rocky zones, rivers, ponds, and puddles
Gonghu Bay	6	Lakeshore rocky zones, rivers, ponds, streams, and puddles

### Sampling design and implementation

To characterize freshwater mollusk distribution, a stratified random design was applied, encompassing lake shores, inflowing rivers/tributaries, ponds, and creeks. Site selection considered substrate (sand, mud, gravel), hydrodynamics (stagnant, slow, fast flow), aquatic vegetation (emergent, submerged, floating-leaved), and disturbance gradients (farmland-suburban-near-natural). Each site was geo-referenced and photographed. In total, 48 sites were surveyed, including 43 sites sampled using quantitative methods and 5 sites sampled using straw mat trapping [19], which was employed where dense vegetation, coarse substrates, or deep mud limited standard sampling. At each straw mat site, two mats (0.1 m^2^ each, spaced 10 to 20 m apart) were deployed and retrieved after 10 days. Samples were washed, sieved through a 0.5-mm mesh, sorted, and identified, with the data incorporated equally into subsequent analyses. Quantitative sampling was conducted using D-frame nets (0.3 m wide, 40 mesh nylon) [20] and Peterson grab samplers (0.0625 m^2^). In wadeable waters, 12 0.09 m^2^ subsamples (total 1.08 m^2^) were collected per site, whereas two parallel grab samples were taken in lake habitats. Lakeshores and ponds were sampled using D-net sweeps combined with hand collection in vegetated zones, and straw mats were applied at selected sites. Slow-flowing rivers with fine substrates were sampled using replicated Peterson grabs, while fast-flowing, coarse-substrate rivers and creeks were surveyed by D-net kick sampling with hand collection beneath cobbles. Sampling effort was standardized by distance, duration, grab number, or mat area to ensure comparability across habitats.

### Sample processing and quality control

All field samples were labeled with Site ID, habitat type, sampling date, and collection method. Within 24 h of collection, samples were washed, sieved through a 0.5-mm mesh, and processed, with straw mat samples washed prior to sieving. Mollusks were sorted under a stereomicroscope, counted, and identified to species level, and shell morphometric measurements were taken when necessary using calipers with a precision of 0.01 mm. Mollusk abundance was standardized and expressed as density (ind·m^−2^). Representative vouchers were preserved in 75% ethanol and DNA subsamples in ≥ 95% ethanol. All data were double entered, with at least 10% of records randomly cross-checked to ensure accuracy. Voucher specimens were cataloged and archived at the Wuxi Center for Disease Control and Prevention (Binhu District, Jiangsu Province). To minimize cross-contamination relevant to parasitological surveillance, all sampling equipment, including nets, grab samplers, trays, and forceps, was disinfected, rinsed, and air-dried between sites. Samples were placed in clean, clearly labeled containers, and disposable gloves were replaced at each site to maintain sample independence and data comparability.

### Literature search and selection

A systematic search was conducted in PubMed (MEDLINE), Web of Science Core Collection, and CNKI (China National Knowledge Infrastructure) up to September 4, 2025, without restriction on starting year. Both published and online-first articles were considered. Search strategies combined core taxonomic and parasitological terms using Boolean logic, including “mollusk(s),” “snail,” “gastropod,” “bivalv*” with “parasite” and “intermediate host” (including “first/second intermediate host” and “host-parasite relations”). Chinese search terms included “mollusk/贝类/螺类” AND “parasite/寄生虫” AND “intermediate host/中间宿主” (including “first intermediate host/第一中间宿主” and “second intermediate host/第二中间宿主”). Targeted searches of key genera or species (e.g., *Radix, Pomacea*) were conducted, and MeSH terms (Mollusca, Parasites, Host-Parasite Relations) were applied in PubMed.

Inclusion criteria were: (i) freshwater mollusks (gastropods or bivalves) as study organisms; (ii) explicit evidence of intermediate host status or infection (natural infection, experimental infection, or molecular detection); (iii) no geographic restriction, with emphasis on China and the Yangtze River Basin; (iv) peer-reviewed original articles or reviews/systematic reviews; (v) publications in English or Chinese. Exclusion criteria included: (i) marine or estuarine studies; (ii) studies not addressing intermediate host status, such as those focused solely on physiology, toxicology, or methodology; (iii) conference abstracts, editorials, notes, or preprints lacking critical data; (iv) inaccessible or incomplete full texts.

Literature screening was independently conducted by two reviewers through title and abstract screening, followed by full-text assessment, with disagreements resolved by a third reviewer. Duplicates were removed using reference management software. Data extraction included host species (italicized binomial nomenclature), parasite species, intermediate host stage, type of evidence, geographic location, sample size/infection prevalence, and ecological information (habitat, feeding traits). All extracted data were cross-checked by two reviewers to ensure accuracy and consistency.

### Species identification, recording, and analysis of density and dominance

Collected snails were identified based on shell morphology (apex, color, proportions) and operculum characteristics (shape, color, texture) [21, 22]. Specimens were photographed using a stereomicroscope (OLYMPUS SZ61) and a smartphone (Mate 30E Pro 5G). For each site, the number of species, individuals, and total specimens was recorded. Mollusks collected using the straw mat trapping method were included only in species richness analyses and excluded from abundance, density, and dominance calculations.

For D-frame net sampling, each quadrat covered 0.09 m^2^, with 12 subsamples per site (total 1.08 m^2^), and density was calculated as the number of individuals per unit area. For Peterson grab sampling, each grab represented 0.0625 m^2^, with two replicates per site, and density was calculated as the total number of individuals divided by the total sampled area. All density values were expressed as individuals per square meter (ind·m^−2^). Dominant species in mollusk communities were determined using the dominance index (Y), calculated as Y = (ni/N) × fi, where ni is the abundance of species i, N denotes the total abundance of all species, and fi is the occurrence frequency (number of sites where species i was detected/total number of sites, 0–1). A species was considered present at a site if detected in any subsample or grab. When calculated at different spatial scales (e.g., bay or entire lake), the denominator corresponded to the number of sites within that scale. Unless otherwise stated, species with Yi ≥ 0.02 were classified as dominant (Table 1).

Community Analysis: Each sampling site was treated as the minimum statistical unit. Alpha diversity indices, including Shannon and Simpson indices, were calculated for each site and summarized at the bay scale (Zhushan Bay, Meiliang Bay, and Gonghu Bay) using medians and interquartile ranges (median [IQR]). When normality assumptions were met, means ± standard deviations were additionally reported. Differences among bays were evaluated using the Kruskal-Wallis rank-sum test, with overall significance reported without post hoc pairwise comparisons.

### Mollusk-parasite relationship network analysis

A bipartite “mollusk-parasite” network was constructed to assess potential public health risks of medicinal mollusks in the northern Lake Taihu wetland. Parasite species associated with mollusk hosts were identified from published literature and field monitoring data, followed by data cleaning and deduplication in R. The curated dataset was imported into Cytoscape 3.10.0 to construct the network, which included 18 mollusk species and 21 parasite species as nodes. Node size was scaled by degree to represent host-parasite connectivity, and node color distinguished mollusks from parasites to highlight species with high structural centrality.

### Diversity indices and statistical analysis

The Shannon and Simpson indices were calculated using the vegan package in R (v4.3.1) to assess the α-diversity level of the community. Differences in diversity among lake bays were tested using the Kruskal-Wallis rank-sum test, with statistical significance set at *p* < 0.05.

Descriptive statistical analysis was performed using SPSS 26.0. The proportions of mollusk families were summarized, and 95% confidence intervals (CIs) were estimated using the Wilson method implemented in the binom package in R.

### Pielou's evenness index analysis

Pielou's evenness index (J′) was calculated from the species abundance matrix using the vegan R package (v2.6–4) to assess the uniformity of individual distribution among species. The index was defined as the ratio of the Shannon diversity index (H′) to species richness (S), with H′ = -Σ (pᵢ × ln pᵢ), where pᵢ is the relative abundance of species i; Pielou's index J′ = H′/ln(S). J′ values were calculated for each sampling site, and differences in evenness among bays were evaluated using the Kruskal-Wallis rank-sum test. Results were visualized using boxplots.

### PCoA based on Bray-Curtis distance

To evaluate differences in community composition among the three bays, a Bray-Curtis distance matrix was calculated from the species abundance matrix, followed by principal coordinate analysis (PCoA) using the cmdscale function in the vegan R package. The first two axes (PCoA1 and PCoA2) and their explained variances were used to depict patterns of community differentiation. Visualization was performed with the ggplot2 R package (v3.4.4), with points representing samples, colors distinguishing bay categories, and 95% confidence ellipses illustrating clustering trends.

### Spatial distribution patterns and hotspot analysis

Spatial hotspots were identified using the Getis-Ord Gi* statistic. A 500-m Euclidean distance threshold was applied as the spatial weight, selected to match the average nearest-neighbor distance among sampling sites and to align with commonly used settings in lake ecosystem and benthic community studies. Mollusk densities (ind·m^−2^) were used as input data, and Gi* values were calculated at the site-species level to identify hotspot locations and frequencies. All medicinal mollusk species were treated equally, without weighting by body size, ecological niche, or public health relevance.

Significance was assessed at *α* = 0.05, with *Z* > 1.96 indicating hotspots and *Z* < − 1.96 indicating cold spots. Results were visualized through spatial distribution maps and bar plots to illustrate significance levels and local clustering patterns. Basemap data were obtained from the National Geoinformation Service Platform, using GCS_WGS_1984 as the geographic coordinate system and WGS_1984_UTM_Zone_49N as the projected coordinate system.

## Results

### Species identification

Specimens collected during the survey were identified based on standard morphological characteristics following authoritative taxonomic references for Chinese freshwater mollusks, including Bogatov & Prozorova (2017) [23] for bivalves and Liu et al. (1979) [21] for gastropods. In total, 2 classes, 5 orders, 10 families, 13 genera, and 20 species were identified, of which 16 species are medicinal mollusks. These species include *Radix swinhoei*, *R. plicatula*, *Polypylis hemisphaerula*, *Gyraulus convexiusculus*, *Hippeutis cantori*, *H. umbilicalis*, *P. canaliculata*, *Alocinma longicornis*, *Parafossarulus eximius*, *P. striatulus*, *S. ningpoensis*, *Sinotaia aeruginosa*, and *S. purificata*, (*S. quadrata*, *Limnoperna lacustri*, and *Corbicula fluminea* (Figure S1). Gastropods accounted for 16 species, belonging to 2 orders, 7 families, and 10 genera. Among them, the family Planorbidae was the most represented, comprising five species (31.3%, 95% CI 14.6–53.4%, estimated using the Wilson method). This was followed by the families Viviparidae and Ancylidae, each with three species (18.8%, 95% CI 5.3–43.7%), and the family Pomatiidae with two species (12.5%, 95% CI 2.2–34.3%). The families Bithyniidae, Amnicolidae, and Hydrobidae were each represented by a single species (6.3%, 95% CI 0.3–28.7%). The bivalve class comprised four species belonging to three orders, three families, and three genera. Corbiculidae was the most represented family, with two species (50.0%, 95% CI 15.0–85.0%), followed by Mytilidae and Unionidae, each with one species (25.0%, 95% CI 4.6–69.9%). Collectively, these 20 mollusk species are reported intermediate hosts for 35 parasite species, playing roles in the transmission of multiple parasitic diseases. Representative parasites include *Fasciola hepatica*, *Echinostoma revolutum*, *Echinostoma hortense*, *Echinoparyphium*, *Euparyphium ilocanum*, *Angiostrongylus cantonensis*, *Trichobilharzia paoi*, *Clonorchis sinensis*, and *Paragonimus westermani* (Table 2).

**Table 2:** Species identification, specimen counts, and identification of intermediate host species capable of transmitting human parasites from medicinal mollusks collected on the northern shore of Lake Taihu wetlands

Species	Parasite host	No. of specimens from different sampling sites (ID)									
		Zhushan Bay					Meiliang Bay				
		No. of sites	Frequency	Cumulative count	Cumulative percentage	Density ± SD (individuals/m^2^)	No. of sites	Frequency	Cumulative count	Cumulative percentage	Density ± SD (individuals/m^2^)
*Gastropoda*											
* Lymnaeidae*		0	0.00%	0	0.00%	0 ± 0	8	16.28%	14	0.87%	27.11 ± 3.57
* Radix swinhoei*	1, 2, 3, 4, 5, 6, 7, 8, 9, 10, 11, 12, 13, 14, 15, 16	0	0.00%	0	0.00%	0 ± 0	7	16.28%	13	0.81%	26.19 ± 3.56
* R. plicatula*	1, 2, 3, 4, 7, 11, 17, 20, 22	0	0.00%	0	0.00%	0 ± 0	1	2.33%	1	0.06%	0.93 ± 0.21
* Planorbidae*		6	13.95%	43	2.68%	46.89 ± 8.57	3	6.98%	5	0.31%	11.7 ± 1.86
* Polypylis hemisphaerula*	2, 4, 6, 20, 21, 22	1	2.33%	1	0.06%	8 ± 1.94	0	0.00%	0	0.00%	0 ± 0
* Gyraulus albus*		1	2.33%	1	0.06%	0.93 ± 0.22	0	0.00%	0	0.00%	0 ± 0
* G. convexiusculus*	2, 4, 5, 19, 20, 21, 22, 23, 25, 26	0	0.00%	0	0.00%	0 ± 0	1	2.33%	3	0.19%	2.78 ± 0.62
* Hippeutis cantori*	2, 4, 21, 22	3	6.98%	3	0.19%	2.78 ± 0.36	2	4.65%	2	0.12%	8.93 ± 1.79
* H. umbilicalis*	4, 5, 21, 23	1	2.33%	38	2.37%	35.19 ± 8.53	0	0.00%	0	0.00%	0 ± 0
* Ampullariidae*		5	11.63%	15	0.93%	13.89 ± 1.85	4	9.30%	35	2.18%	32.41 ± 4.2
* Pomacea canaliculata*	6	5	11.63%	15	0.93%	13.89 ± 1.85	4	9.30%	35	2.18%	32.41 ± 4.2
Bithyniidae		16	27.91%	109	6.79%	207.04 ± 28.7	12	20.93%	23	1.43%	113.26 ± 16.54
* Alocinma longicornis*	24, 25, 26, 32	4	9.30%	36	2.24%	104.07 ± 19.57	2	4.65%	2	0.12%	8.93 ± 1.79
* Parafossarulus eximius*	31	4	9.30%	15	0.93%	13.89 ± 1.57	7	16.28%	12	0.75%	60.63 ± 8.65
* P. striatulus*	4, 24, 25, 26, 27, 28, 29, 30, 31, 32	8	18.60%	58	3.61%	89.07 ± 10.24	3	6.98%	9	0.56%	43.7 ± 8.92
* Plenroseridae*		0	0.00%	0	0.00%	0 ± 0	0	0.00%	0	0.00%	0 ± 0
* Sinotaia ningpoensis*	32, 33	0	0.00%	0	0.00%	0 ± 0	0	0.00%	0	0.00%	0 ± 0
* Stenothyridae*		0	0.00%	0	0.00%	0 ± 0	1	2.33%	1	0.06%	0.93 ± 0.21
* Stenothyra* glabra		0	0.00%	0	0.00%	0 ± 0	1	2.33%	1	0.06%	0.93 ± 0.21
* Viviparidae*		28	37.21%	624	38.88%	1688.41 ± 300	35	44.19%	366	22.80%	727.96 ± 86.78
* Sinotaia aeruginosa*	29	15	34.88%	578	36.01%	1645.81 ± 300.15	18	41.86%	298	18.57%	573.04 ± 67.26
* S. purificata*	Medical mollusk related	7	16.28%	33	2.06%	30.56 ± 4.32	10	23.26%	40	2.49%	121.93 ± 21.28
* S. quadrata*	6	6	13.95%	13	0.81%	12.04 ± 1.2	7	16.28%	28	1.74%	33 ± 4.18
*Bivalvia*											
* Mytilidae*		1	2.33%	1	0.06%	0.93 ± 0.22	1	2.33%	1	0.06%	0.93 ± 0.21
* Limnoperna lacustris*	34, 35	1	2.33%	1	0.06%	0.93 ± 0.22	1	2.33%	1	0.06%	0.93 ± 0.21
* Corbiculidae*		3	6.98%	10	0.62%	9.26 ± 1.27	0	0.00%	0	0.00%	0 ± 0
* Cosmioconcha nitens*		1	2.33%	4	0.25%	3.7 ± 0.9	0	0.00%	0	0.00%	0 ± 0
* C. fluminea*	2	2	4.65%	6	0.37%	5.56 ± 0.98	0	0.00%	0	0.00%	0 ± 0
* Unionidae*		2	4.65%	2	0.12%	1.85 ± 0.31	0	0.00%	0	0.00%	0 ± 0
Unio douglasiae		2	4.65%	2	0.12%	1.85 ± 0.31	0	0.00%	0	0.00%	0 ± 0

Species	No. of specimens from different sampling sites (ID)									
	Gonghu Bay					Total				
	No. of sites	Frequency	Cumulative count	Cumulative percentage	Density ± SD (individuals/m^2^)	No. of sites	Frequency	Cumulative count	Cumulative percentage	Density ± SD (individuals/m^2^)
*Gastropoda*										
* Lymnaeidae*	1	2.33%	2	0.12%	1.85 ± 0.76	9	18.60%	16	1.00%	91.86 ± 7.11
* Radix swinhoei*	1	2.33%	2	0.12%	1.85 ± 0.76	8	18.60%	15	0.93%	28.04 ± 2.49
* R. plicatula*	0	0.00%	0	0.00%	0 ± 0	1	2.33%	1	0.06%	0.93 ± 0.14
* Planorbidae*	0	0.00%	0	0.00%	0 ± 0	9	20.93%	48	2.99%	142.1 ± 12.91
* Polypylis hemisphaerula*	0	0.00%	0	0.00%	0 ± 0	1	2.33%	1	0.06%	8 ± 1.22
* Gyraulus albus*	0	0.00%	0	0.00%	0 ± 0	1	2.33%	1	0.06%	0.93 ± 0.14
* G. convexiusculus*	0	0.00%	0	0.00%	0 ± 0	1	2.33%	3	0.19%	2.78 ± 0.42
* Hippeutis cantori*	0	0.00%	0	0.00%	0 ± 0	5	11.63%	5	0.31%	11.7 ± 1.24
* H. umbilicalis*	0	0.00%	0	0.00%	0 ± 0	1	2.33%	38	2.37%	35.19 ± 5.37
* Ampullariidae*	0	0.00%	0	0.00%	0 ± 0	9	20.93%	50	3.12%	129.53 ± 10.12
* Pomacea canaliculata*	0	0.00%	0	0.00%	0 ± 0	9	20.93%	50	3.12%	46.3 ± 3.1
Bithyniidae	8	13.95%	74	4.61%	75.59 ± 7.49	36	62.79%	206	12.83%	1052.85 ± 77.71
* Alocinma longicornis*	0	0.00%	0	0.00%	0 ± 0	6	13.95%	38	2.37%	113 ± 12.47
* Parafossarulus eximius*	2	4.65%	2	0.12%	1.85 ± 0.48	13	30.23%	29	1.81%	76.37 ± 6.02
* P. striatulus*	6	13.95%	72	4.49%	73.74 ± 7.51	17	39.53%	139	8.66%	206.52 ± 9.69
* Plenroseridae*	1	2.33%	1	0.06%	0.93 ± 0.38	1	2.33%	1	0.06%	3.3 ± 0.27
* Sinotaia ningpoensis*	1	2.33%	1	0.06%	0.93 ± 0.38	1	2.33%	1	0.06%	0.93 ± 0.14
* Stenothyridae*	0	0.00%	0	0.00%	0 ± 0	1	2.33%	1	0.06%	2.2 ± 0.23
* Stenothyra* glabra	0	0.00%	0	0.00%	0 ± 0	1	2.33%	1	0.06%	0.93 ± 0.14
* Viviparidae*	14	13.95%	279	17.38%	647.41 ± 167.28	77	95.35%	1269	79.07%	8246.45 ± 603.96
* Sinotaia aeruginosa*	5	11.63%	155	9.66%	334.52 ± 82.59	38	88.37%	1031	64.24%	2553.37 ± 195.44
* S. purificata*	6	13.95%	119	7.41%	308.26 ± 85.6	23	53.49%	192	11.96%	460.74 ± 36.92
* S. quadrata*	3	6.98%	5	0.31%	4.63 ± 1.08	16	37.21%	46	2.87%	49.67 ± 2.97
*Bivalvia*										
* Mytilidae*	0	0.00%	0	0.00%	0 ± 0	2	4.65%	2	0.12%	5.26 ± 0.41
* Limnoperna lacustris*	0	0.00%	0	0.00%	0 ± 0	2	4.65%	2	0.12%	1.85 ± 0.2
* Corbiculidae*	0	0.00%	0	0.00%	0 ± 0	3	6.98%	10	0.62%	20.62 ± 2.24
* Cosmioconcha nitens*	0	0.00%	0	0.00%	0 ± 0	1	2.33%	4	0.25%	3.7 ± 0.56
* C. fluminea*	0	0.00%	0	0.00%	0 ± 0	2	4.65%	6	0.37%	5.56 ± 0.63
* Unionidae*	0	0.00%	0	0.00%	0 ± 0	2	4.65%	2	0.12%	4.21 ± 0.45
Unio douglasiae	0	0.00%	0	0.00%	0 ± 0	2	4.65%	2	0.12%	1.85 ± 0.2

1. *Fasciola hepatica*; 2. *Echinostoma revolutum*; 3. *E. hortense*; 4. *E. recurvatum*; 5. *E. ilocanum*; 6. *Angiostrongylus cantonensis*; 7. *Trichobilharzia paoi*; 8. *Trichobilharzia physella*; 9. *Trichobilharzia ocellata*; 10. *Trichobilharzia gigantica*; 11. *Orientobilharzia turkestanicum*; 12. *Pseudobilharziella yokogawai*; 13. *Cercaria okiensis*; 14. *Plagiorchis muris*; 15. *Diplostolmum niedashui*; 16. *Diplostolmum hupensis*; 17. *Orientobilharzia cheni*; 18. *Sanguinicola lungjiangensis*; 19. *Echinostoma malayanum*; *20. Echinostoma cinetorchis*; *21. Fasciolopsis buski*; 22. *Echinostoma miyagawai*; 23. *Echinostoma ilocanum*; 24. *Haplorchis yokogawaii*; 25. *Haplorchis pumilio*; 26. *Haplorchis taichui*; 27. *Metorchis orientalis*; 28. *Metorchis taiwanensis*; 29. *Echinochasmus perfoliatus*; 30. *Echinochasmus japonicus*; 31. *Asymphylodora japonica*; 32. *Clonorchis sinensis*; 33. *Paragonimus westermani*; 34. *Parabucephalopsis prosorchis*; 35. *Dollfustrema vaneyi*. Snails collected using the straw curtain trapping method were excluded from density calculations

### Literature screening results and construction of host-parasite associations

As of September 2025, a total of 424 records had been retrieved from PubMed (*n* = 179), Web of Science Core Collection (*n* = 236), and CNKI (*n* = 9), with no additional records identified through other sources (*n* = 0). After duplicate removal, 187 records remained for title and abstract screening, of which 168 were excluded for failing to meet the inclusion criteria. Nineteen articles were subsequently assessed in full text, and 15 studies met the eligibility criteria and were included in the construction of the mollusk-parasite host association database. From these studies, 92 host-parasite pairs were extracted, comprising 18 mollusk species and 21 parasite species, which were used to construct the bipartite network. The complete literature screening process is shown in Figure S2.

### Species abundance, distribution, and dominance

A total of 1605 mollusk specimens were collected between 2022 and 2023 (Table 2). Among them, Meiliang Bay yielded 804 specimens, accounting for 50.09% of the total (804/1605), and included mollusks from 7 families and 15 genera. Zhushan Bay contributed 445 specimens (27.73%, 445/1605), representing 7 families and 13 species, while Gonghu Bay had the lowest abundance and diversity, with 356 specimens (22.18%, 356/1605) from 4 families and 7 species (Fig. 3).

**Fig. 2:**
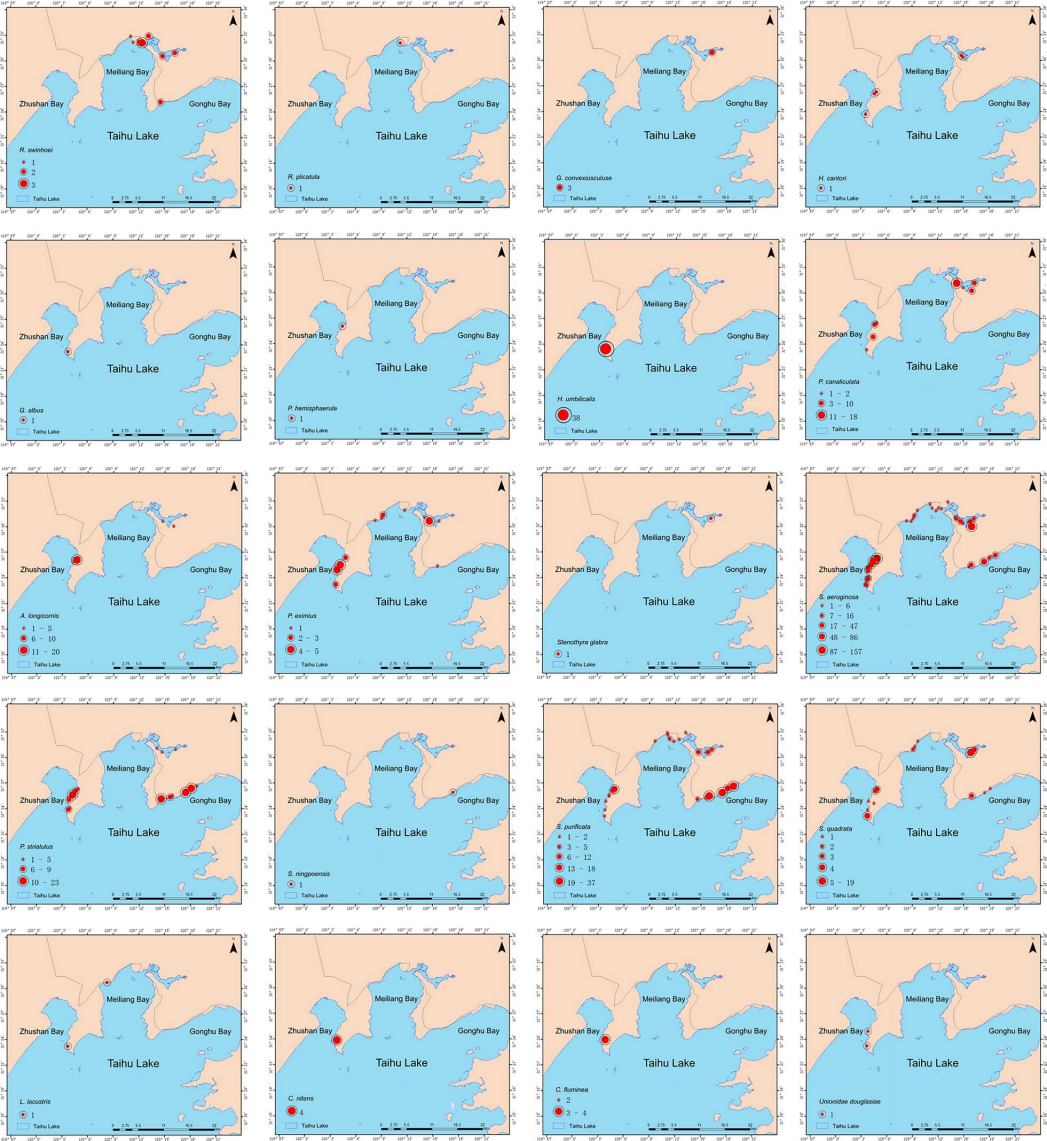
Spatial distribution of medicinal mollusk abundance in the Lake Taihu wetland

Overall, 10 families and 20 mollusk species were identified across the three bays. Zhushan Bay exhibited the highest species richness, with 7 families and 15 species, followed by Meiliang Bay with 7 families and 13 species, and Gonghu Bay with 4 families and 7 species. The family Planorbidae contained the most species (*n* = 5), whereas Viviparidae showed the highest frequency, total abundance, cumulative proportion, and mean density (8246.45 ± 603.96). Common species, including *P. eximius*, *S. aeruginosa*, and *S. purificata*, were widely distributed across the northern Lake Taihu wetland. Based on dominance index values (*Y* ≥ 0.02), *S. aeruginosa*, *S. purificata*, and *P. striatulus* were identified as dominant species. Among these, *S. aeruginosa* was the dominant species across all three bays. Additionally, *S. purificata* was dominant in Meiliang Bay, while both *S. purificata* and *P. striatulus* were dominant in Zhushan Bay. The highest density areas for *S. aeruginosa* were primarily in Meiliang Bay and Zhushan Bay, while *S. purificata* was most abundant in Gonghu Bay, and *P. striatulus* had its highest distribution in Zhushan Bay and Gonghu Bay (Fig. 2) (Table 3).

**Table 3:** Dominant medicinal mollusk species in the northern wetlands of Lake Taihu

Species	Overall	River name		
		Zhushan Bay	Meiliang Bay	Gonghu Bay
	Dominance index (Y)	Dominance index (Y)	Dominance index (Y)	Dominance index (Y)
*R. swinhoei*	0.0017	< 0.0001	0.0048	0.0001
*R. plicatula*	< 0.0001	< 0.0001	0.0001	< 0.0001
*P. hemisphaerula*	< 0.0001	< 0.0001	< 0.0001	< 0.0001
*G. albus*	< 0.0001	< 0.0001	< 0.0001	< 0.0001
*G. convexiusculus*	< 0.0001	< 0.0001	0.0002	< 0.0001
*H. cantori*	0.0004	0.0003	0.0002	< 0.0001
*H. umbilicalis*	0.0006	0.0011	< 0.0001	< 0.0001
*P. canaliculata*	0.0065	0.0022	0.0073	< 0.0001
*A. longicornis*	0.0033	0.0042	0.0002	< 0.0001
*P. eximius*	0.0055	0.0017	0.0044	0.0003
*P. striatulus*	0.0342	0.0134	0.0014	0.0282
*S. ningpoensis*	< 0.0001	< 0.0001	< 0.0001	0.0001
*S. glabra*	< 0.0001	< 0.0001	0.0001	< 0.0001
*S. aeruginosa*	0.5677	0.2508	0.2803	0.0506
*S. purificata*	0.0640	0.0067	0.0209	0.0466
*S. quadrata*	0.0107	0.0023	0.0102	0.0010
*L. lacustris*	0.0001	< 0.0001	0.0001	< 0.0001
*C. nitens*	0.0001	0.0001	< 0.0001	< 0.0001
*C. fluminea*	0.0002	0.0003	< 0.0001	< 0.0001
*U. douglasiae*	0.0001	0.0001	< 0.0001	< 0.0001

*R. swinhoei*: *Radix swinhoei*; *R. plicatula: Radix plicatula*; *P. hemisphaerula: Polypylis hemisphaerula*; *G. albus: Gyraulus albus*; *G. convexiusculus: Gyraulus convexiusculus*; *H. cantori: Hippeutis cantori*; *H. umbilicalis: Hippeutis umbilicalis*; *P. canaliculata: Pomacea canaliculata*; *A. longicornis: Alocinma longicornis*; *P. eximius: Parafossarulus eximius*; *P. striatulus: Parafossarulus striatulus*; *S. ningpoensis: Sinotaia ningpoensis*; *S. glabra: Stenothyra glabra*; *S. aeruginosa: Sinotaia aeruginosa*; *S. purificata: Sinotaia purificata*; *S. quadrata: Sinotaia quadrata*; *L. lacustris: Limnoperna lacustris*; *C. nitens: Corbicula nitens*; *C. fluminea: Corbicula fluminea*; *U. douglasiae: Unio douglasiae*

### Diversity index analysis

**Fig. 3:**
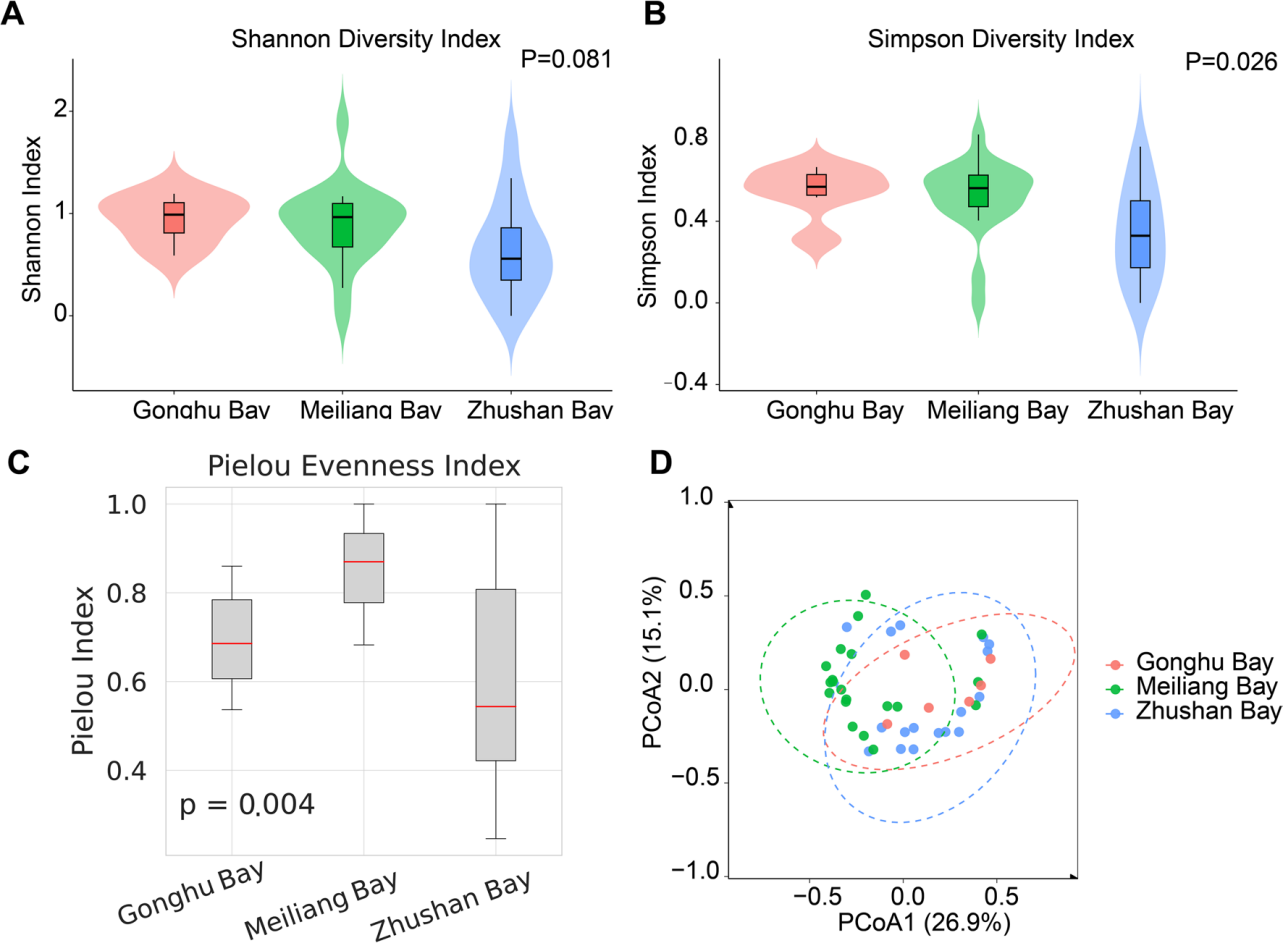
Comparison of α-diversity indices for mollusk communities across three major bays. **A** The Shannon index shows differences in community diversity trends; **B** the Simpson index reflects differences in community evenness. Violin plots in the figure display the distribution density at each sampling site in the bays, while box plots represent the median and interquartile range; **C** Pielou’s evenness index of mollusk communities across different bays. **D** PCoA based on Bray-Curtis distance showing community differentiation among bays

The Shannon index did not differ significantly among the three bays (*χ*^2^ = 5.038, df = 2, *p* = 0.081), whereas the Simpson index showed significant variation (*χ*^2^ = 7.314, df = 2, *p* = 0.026), reflecting differences in community evenness and dominance (Fig. 3A, B). Pielou's evenness index was significantly lower in Zhushan Bay than in Gonghu Bay and Meiliang Bay, suggesting a stronger dominance effect in Zhushan Bay (Fig. 3C). PCoA based on Bray-Curtis distances revealed clear separation of community structures among the three bays. This pattern was consistent with results from weighted and unweighted UniFrac analyses, which further confirmed differences in both species abundance and phylogenetic composition among bays (Fig. 3D).

### Hotspot analysis

**Fig. 4:**
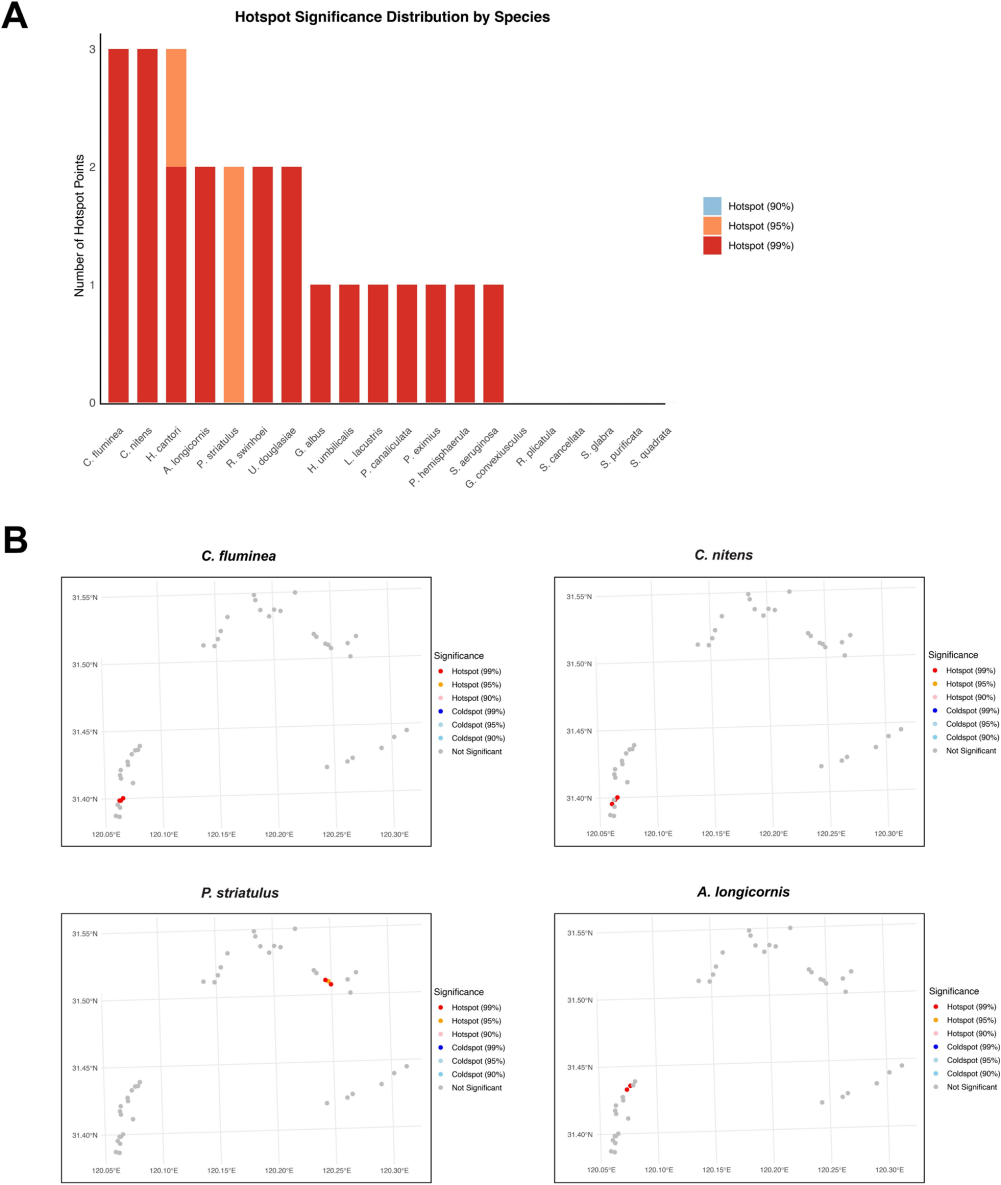
Statistical number and spatial distribution characteristics of significant hotspots in medicinal mollusks. **A** The number of hotspot sampling points for each species at the 99%, 95%, and 90% significance levels. The stacked bar chart illustrates the degree of local aggregation for 19 mollusk species at each confidence level. **B** Spatial distribution maps of the top four species with the highest hotspot numbers (*Corbicula fluminea*, *C. nitens*, *Hippeutis cantori*, *Alocinma longicornis*) in the sampling area

Hotspot analysis of 19 medicinal mollusk species in the northern Lake Taihu wetland was conducted using the Getis-Ord Gi* statistic, revealing significant spatial aggregation at the 99%, 95%, and 90% confidence levels (Table 4). The strongest aggregation was observed for *C. fluminea*, *C. nitens*, and *H. cantori*, each exhibiting three hotspots at the 99% confidence level. *Alocinma longicornis* and *P. striatulus* showed moderate aggregation, with two hotspots detected at the 99% and 95% levels, respectively (Fig. 4A). Despite relatively high overall densities, species such as *S. aeruginosa* and *P. canaliculata* formed only localized hotspots, indicating widespread but less concentrated distributions. In contrast, *R. plicatula* and *S. cancellata* showed no significant hotspots, likely reflecting low density or highly dispersed patterns. Further analysis of the four species with the highest hotspot numbers (*C. fluminea*, *C. nitens*, *H. cantori*, *A. longicornis*) confirmed that most high-confidence hotspots were concentrated in Zhushan Bay, suggesting this region represents a priority zone for surveillance and control (Fig. 4B). Average and maximum densities for all species are provided in Table S2.

**Table 4:** Number of hotspot and coldspot sampling sites for each medicinal mollusk species in the Lake Taihu region at different significance levels

Species	Hotspot (99%)	Hotspot (95%)	Hotspot (90%)	Coldspot (99%)	Coldspot (95%)	Coldspot (90%)	Not significant
*A. longicornis*	2	NA	0	0	0	0	41
*C. fluminea*	3	NA	0	0	0	0	40
*C. nitens*	3	NA	0	0	0	0	40
*G. albus*	1	NA	0	0	0	0	42
*G. convexiusculus*	NA	NA	0	0	0	0	43
*H. cantori*	2	1	0	0	0	0	40
*H. umbilicalis*	1	NA	0	0	0	0	42
*L. lacustris*	1	NA	0	0	0	0	42
*P. eximius*	1	NA	0	0	0	0	42
*P.striatulus*	NA	2	0	0	0	0	41
*P. hemisphaerula*	1	NA	0	0	0	0	42
*P.canaliculata*	1	NA	0	0	0	0	42
*R. plicatula*	NA	NA	0	0	0	0	43
*R. swinhoei*	2	NA	0	0	0	0	41
*S.cancellata*	NA	NA	0	0	0	0	43
*S.aeruginosa*	1	NA	0	0	0	0	42
*S. purificata*	NA	NA	0	0	0	0	43
*S. quadrata*	NA	NA	0	0	0	0	43
*S. glabra*	NA	NA	0	0	0	0	43
*U. douglasiae*	2	NA	0	0	0	0	41

*A. longicornis: Alocinma longicornis; C. fluminea: Corbicula fluminea; C. nitens: Corbicula nitens; G. albus: Gyraulus albus; G. convexiusculus: Gyraulus convexiusculus; H. cantori: Hippeutis cantori; H. umbilicalis: Hippeutis umbilicalis; L. lacustris: Limnoperna lacustris; P. eximius: Parafossarulus eximius; P. striatulus: Parafossarulus striatulus; P. hemisphaerula: Polypylis hemisphaerula; P. canaliculata: Pomacea canaliculata; R. plicatula: Radix plicatula; R. swinhoei: Radix swinhoei; S. cancellata: Semisulcospira cancellata; S. aeruginosa: Sinotaia aeruginosa; S. purificata: Sinotaia purificata; S. quadrata: Sinotaia quadrata; S. glabra: Stenothyra glabra; U. douglasiae: Unio douglasiae*. Hotspots (99%), (95%), and (90%) indicate the number of hotspot sites at the corresponding confidence levels. Coldspot refers to coldspot sites at the same significance levels. 'Not significant' represents the number of sampling sites without statistically significant spatial clustering

### Mollusk-parasite network analysis

**Fig. 5:**
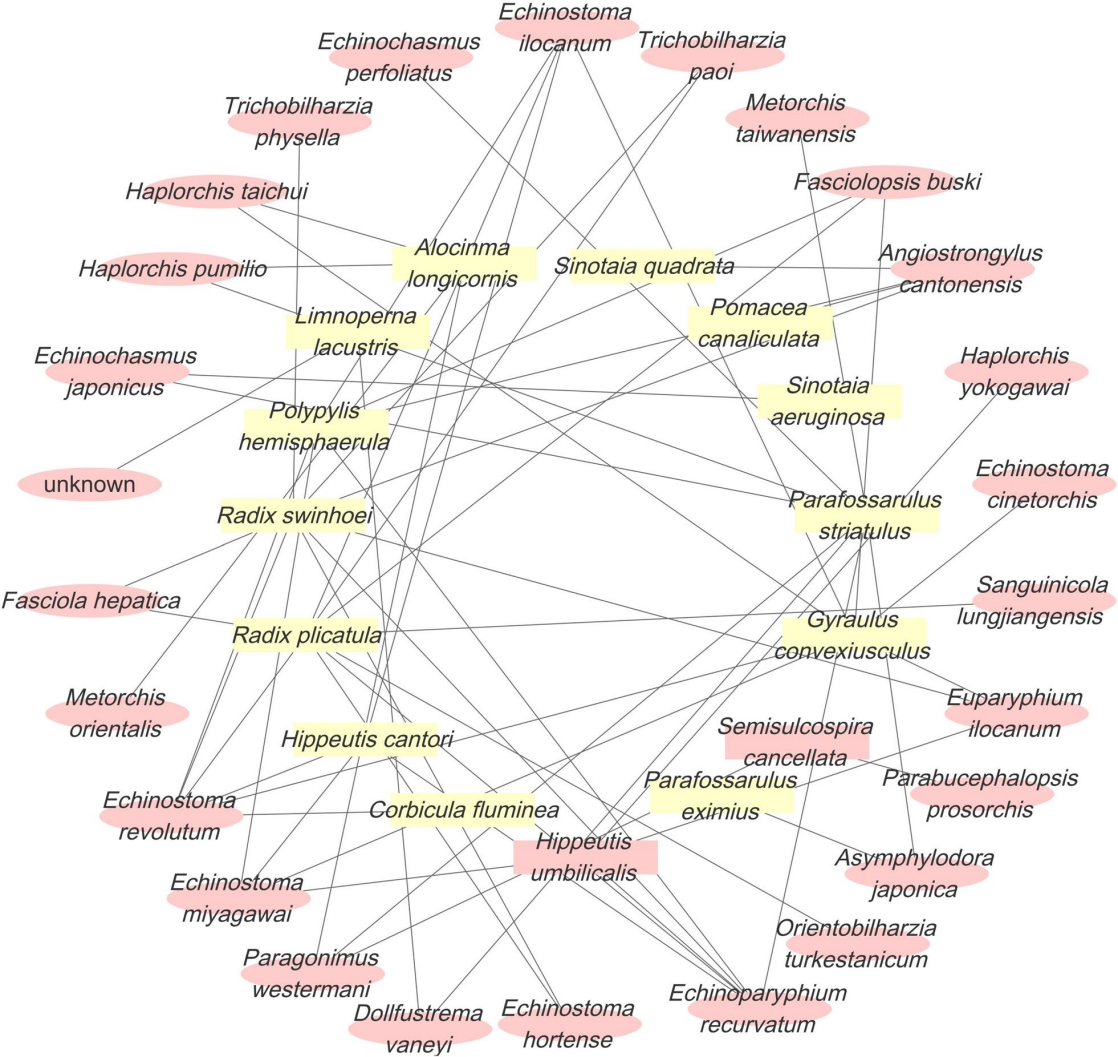
Mollusk-parasite network. Pink ovals represent parasites, yellow squares represent mollusks, and gray lines indicate parasite-host relationships. The network consists of 39 nodes and 92 connections. Core hosts: *Radix plicatula* (degree 9) and *R. swinhoei* (degree 8). High-adaptation parasites: *Echinostoma revolutum*, *Angiostrongylus cantonensis*, and *Fasciolopsis buski* (connected to ≥ 7 mollusk species)

The bipartite “mollusk-parasite” network constructed in this study comprised 39 nodes and 92 parasite-host links, illustrating complex associations between mollusk species and their reported parasites based on the literature and field monitoring data. Among the mollusk hosts, *R. plicatula* (connected with 9 parasite species) and *R. swinhoe*i (connected with 8 parasite species) exhibited the highest degree values and were positioned at the central region of the network. Several other mollusks, including *H. cantori, P. hemisphaerula*, and *G. convexiusculus*, also showed relatively high degrees, indicating multiple recorded parasite associations (Fig. 5). From the parasite perspective, species such as *E. revolutum*, *A. cantonensis*, and *Fasciolopsis buski* were each linked to seven or more mollusk species, reflecting broad host ranges within the compiled dataset. Overall, the network visualization enabled the identification of mollusk species with high connectivity and parasite taxa associated with multiple recorded hosts (Table S1).

## Discussion

This study documented 20 mollusk species along the northern shore of Lake Taihu, of which 16 are recognized intermediate hosts of medical relevance. The observed diversity pattern is consistent with surveys from other freshwater systems in China and internationally [24–26], where species such as *P. canaliculata* and *Radix* spp. are widely reported as hosts for trematodes and nematodes [27, 28]. In contrast to these broader patterns, mollusk communities in Lake Taihu exhibited pronounced regional heterogeneity. Zhushan Bay supported fewer species but markedly higher population densities, whereas Meiliang Bay and Gonghu Bay showed more balanced community assemblages. Environmental factors, including water quality, sediment characteristics, and temperature, likely contribute to these spatial differences, consistent with previous studies demonstrating habitat filtering effects on mollusk community structure [29]. By integrating species composition, density distribution, community structure, host-parasite network centrality, and spatial hotspot patterns at a regional scale, this study provides a more systematic and quantitative assessment of medicinal mollusks from both ecological and public health perspectives. This integrative approach enhances the explanatory power of the results and establishes a clearer logical foundation for the subsequent discussion.

The study found that *S. aeruginosa* exhibited the highest density, reaching 2553.37 ± 195.44 ind·m⁻^2^, far exceeding that of other mollusk species. This pattern is consistent with previous reports [30–32] and indicates strong ecological adaptability and the capacity to form high-density populations across diverse aquatic environments. In contrast, *R. swinhoei* and *R. plicatula*, although more species-rich, showed relatively low densities, likely reflecting narrower requirements for water quality and sediment conditions. Variations in water quality, substrate type, and food availability have been shown to shape mollusk distribution and abundance across different bays [33], and similar interactions between species traits and environmental factors have been documented in other wetland ecosystems [34, 35]. Notably, these exceptionally high densities imply that any parasites harbored by *S. aeruginosa* may be disproportionately amplified, thereby increasing infection pressure within the aquatic environment. This characteristic positions *S. aeruginosa* not only as an ecological dominant but also as a potential “risk-amplifying node” from a public health perspective, underscoring the need to prioritize this species in regional parasite surveillance and control strategies.

This study constructed a mollusk-parasite bipartite network and identified close associations between multiple mollusk species and multihost parasites, such as *E. revolutum*, *A. cantonensis*, and *F. buski*. Notably, *R. swinhoei* and *R. plicatula* occupied central positions, reflecting their high connectivity with diverse parasite taxa. Compared with existing studies [36], this research represents the first systematic analysis of ecological relationships between mollusks and parasites along the northern shore of Lake Taihu, underscoring the critical role of freshwater mollusks as intermediate hosts in parasite transmission. Consistent with previous findings, mollusks have been shown to serve as hosts for a wide range of parasites, and areas with high mollusk abundance generally pose increased risks for parasite transmission [37–39]. High-connectivity taxa such as *Radix* spp. act as shared hosts for multiple parasites and may maintain transmission pathways even under environmental disturbance, conferring structural importance within the regional host–parasite system. Accordingly, the network results suggest that surveillance and control efforts should prioritize these core host species, rather than focusing exclusively on high-density taxa, to enable more proactive and effective risk management.

Spatial distribution hotspots of mollusks were analyzed using ArcGIS software, and the results identified Zhushan Bay as a high-risk area for mollusk aggregation. This pattern is consistent with findings from other wetland ecosystems, which indicate that freshwater mollusks commonly exhibit spatial aggregation and that such aggregation zones are frequently associated with elevated risks of parasite transmission [40–42]. For example, studies from the Yangtze River Basin have demonstrated a close association between mollusk community aggregation and parasite transmission dynamics [43]. The identification of spatial hotspots not only depicts the clustering patterns of freshwater mollusks but also reveals the spatial heterogeneity of potential infection risk. The consistently high-confidence hotspots observed in Zhushan Bay across multiple species indicate that this area may function as a key spatial node for parasite amplification and dispersal. Accordingly, hotspot analysis extends beyond descriptive mapping and provides actionable spatial information for regional early-warning systems, targeted field interventions, and resource allocation.

Beyond *S. aeruginosa*, species with high frequency and network connectivity, such as *R. swinhoei* and *R. plicatula*, play pivotal roles in regional freshwater ecosystems. These species are widely distributed across lakes, rice paddies, and stagnant channels in the middle and lower Yangtze River Basin [44], typically inhabiting slow-flowing or stagnant waters with fine sediments (silt or mud). They commonly co-occur with emergent or submerged vegetation and feed primarily on periphyton and detritus in eutrophic environments [45, 46]. Moreover, *Radix* species are well-documented first intermediate hosts for multiple trematodes [47, 48], underscoring their central role in potential zoonotic parasite transmission networks. The bipartite network analysis in this study aligns with these ecological insights, further supporting the prioritization of these species in regional monitoring and surveillance efforts.

This study highlights the complex relationship between mollusk diversity and public health risk. Although the northern shore of Lake Taihu supports high species richness, species differ markedly in density and aggregation. Key hosts such as *S. aeruginosa*, *R. swinhoei*, and *R. plicatula* occupy central positions within the parasite transmission network, indicating that species aggregation and host importance, rather than overall diversity, are primary drivers of public health risk. Spatial analyses further revealed areas of high density and concentrated hotspots, with Zhushan Bay identified as a critical high-risk region. Together, these findings emphasize the need for targeted monitoring and control strategies that prioritize hotspot areas and focus on key host species to more effectively mitigate parasite transmission risk.

The ecological and spatial patterns identified in this study converge on a central insight: medicinal mollusks function simultaneously as ecological nodes and parasite-transmission nodes within freshwater systems, embedding their potential health risks within the broader human-animal-environment interface. Species such as *Radix* and *Parafossarulus* contribute to nutrient cycling and benthic food-web maintenance [49] while also serving as first intermediate hosts for multiple medically important parasites. In East Asia, these species further hold traditional medicinal and dietary value, yet consumption of raw or inadequately processed individuals has been linked to foodborne parasitic infections such as angiostrongyliasis and clonorchiasis [50]. Anthropogenic disturbances, including eutrophication, shoreline modification, and agricultural runoff, can elevate parasite burdens in freshwater habitats [38], and the strong environmental tolerance of these mollusks makes them representative of the ecology-health interface organisms. The baseline information and hotspot identification provided by this study, particularly the high-risk zone in Zhushan Bay, highlight spatial nodes where ecological functioning and parasite amplification intersect, offering direct guidance for monitoring priorities, ecological management, and health-education strategies [51]. By integrating ecological roles, traditional uses, and public health risks, this study more accurately reflects the One Health principle of interdependent human, animal, and environmental health [52, 53] and supports the development of regional early-warning systems for mollusk-borne diseases. Given the continued harvesting and consumption of these species in some areas, future work should establish safer collection, processing, and consumption guidelines within a One Health framework to balance sustainable resource use with effective risk mitigation.

This study has several limitations. First, the relatively short study period limited the characterization of seasonal and long-term dynamics of mollusk communities relevant to parasite transmission. Second, species identification relied primarily on morphological criteria, which, although reliable, may constrain the detection of cryptic or closely related taxa in the absence of molecular confirmation. Third, key environmental parameters, including dissolved oxygen, pH, total nitrogen/total phosphorus, chlorophyll a, sediment grain size, and organic matter, were not measured concurrently, resulting in conservative interpretations of ecological drivers affecting host distribution. Finally, inter-bay comparisons were assessed using only the Kruskal-Wallis test without post hoc pairwise analyses. Future studies should incorporate Dunn's test or paired Wilcoxon rank-sum tests with appropriate multiple-comparison corrections to improve statistical resolution [54].

Future research should adopt an integrated framework across ecological, molecular, and public health dimensions. Ecologically, long-term and stratified monitoring of water quality (TN/TP, DO, temperature, pH, Chl-a) and sediment conditions (grain size, organic matter) is needed to identify key drivers of mollusk distribution [55, 56]. Molecularly, eDNA, metabarcoding, and species-specific qPCR/digital PCR approaches should be employed to improve the detection and surveillance of both parasites and their mollusk hosts. From a public health perspective, integrating ecological and molecular data with human behavioral and clinical information would enable refined risk mapping, early warning, and assessment of interventions, including aquaculture management, buffer zone establishment, and health education [57]. This interdisciplinary integration will support the development of predictive, evaluative, and actionable control strategies for mollusk-borne parasitic diseases.

In conclusion, this study provides a systematic baseline of mollusk community structure and spatial distribution along the northern shore of Lake Taihu and identifies Zhushan Bay as a high-risk spatial hotspot. Within the One Health framework, our findings highlight the ecological functions and traditional uses of medicinal mollusks, as well as their relevance to parasite transmission risk. The baseline information and hotspot identification can directly inform subsequent parasitological surveillance and public health interventions, including indicator-species selection, sampling-site optimization, origin control and purification procedures, spatiotemporal management of harvesting, and risk communication. These applications will help reduce the transmission risk of regional mollusk-borne parasitic diseases while ensuring the sustainable use of traditional medicinal resources.

## Conclusions

This study systematically investigated the species composition, spatial distribution, and public health risks of medicinal mollusks along the northern shore of Lake Taihu. In total, 20 mollusk species were identified, 16 of which are recognized intermediate hosts for parasites. *Sinotaia aeruginosa*, *S. purificata*, and *P. striatulus* were identified as dominant species, with *S. aeruginosa* exhibiting markedly higher density than other taxa, indicating strong ecological competitiveness and elevated transmission potential. Spatial analysis revealed significant aggregation of species such as *A. longicornis*, *C. fluminea*, and *C. nitens* in the Zhushan Bay area, identifying this area as a high-risk zone for parasitic diseases that warrants enhanced monitoring and intervention. In addition, the mollusk-parasite transmission network analysis identified *Radix swinhoei* and *R. plicatula* as key host nodes within the transmission chain, improving the scientific basis and forward-looking capacity of risk assessment. By integrating spatial analysis tools such as ArcGIS with transmission network topology, this study provides a visual and quantitative assessment of mollusk distribution patterns and associated transmission risks. The findings fill a gap in regional ecological and parasitological monitoring and offer practical support for local disease control and ecological management, facilitating the development of more targeted control strategies.

## Supplementary Information

Supplementary Material 1. Figure S1. Images of 20 medicinal mollusk species from the Lake Taihu wetland. 1 (abc). *Sinotaia quadrata*; 2 (abc). *Radix swinhoei*; 3 (abc). *Unio douglasiae*; 4 (abc). *Sinotaia purificata*; 5 (abc). *S. ningpoensis*; 6 (abc). *Limnoperna lacustris*; 7 (abc). *Pomacea canaliculata*; 8 (abc). *Radix plicatula*; 9 (abc). *Sinotaia aeruginosa*; 10 (abc). *Parafossarulus eximius*; 11 (ab). *Corbicula fluminea*; 12 (abc). *Corbicula nitens*; 13 (ab). *Polypylis hemisphaerula*; 14 (ab). *H. umbilicalis*; 15 (ab). *Gyraulus albus*; 16 (ab). *Hippeutis cantori*; 17 (abc). *Stenothyra glabra *(*S. glabra)*; 18 (abc). *Parafossarulus striatulus*; 19 (abc). *Alocinma longicornis*; 20 (ab). *Gyraulus convexiusculus*. Scale bar length: 18 mm

Supplementary Material 2. Figure S2. Literature screening process for identifying key host-parasite associations.

Supplementary Material 3.

## Data Availability

All data generated or analyzed during this study are included in this article and/or its supplementary material files.

## Abbreviations

CI: Confidence interval
DNA: Deoxyribonucleic acid
DO: Dissolved oxygen
GIS: Geographic information system
IQR: Interquartile range
J′: Pielou’s evenness index
PCR: Polymerase chain reaction
qPCR: Quantitative polymerase chain reaction
SPSS: Statistical Package for the Social Sciences
UTM: Universal Transverse Mercator
