# Supplementary figures and images for: Ecological patterns and spatial distribution of medicinal mollusks in a freshwater ecosystem
Source: Parasit Vectors. 2026 Feb 18;19:119. doi: 10.1186/s13071-026-07273-9 (PMC12997700; doi:10.1186/s13071-026-07273-9)

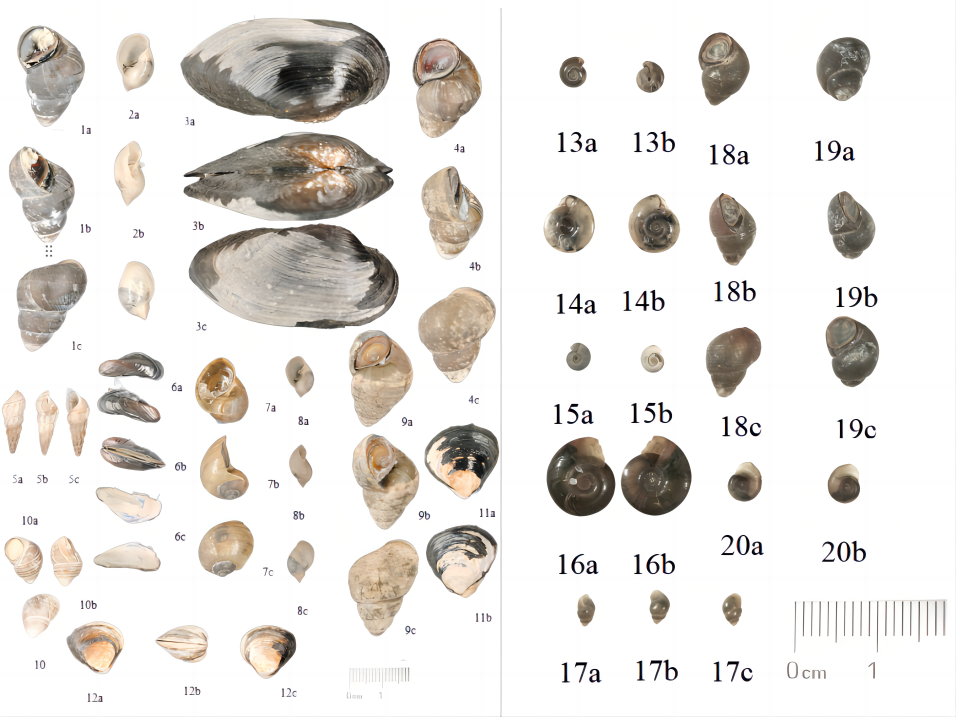

Supplement: Supplementary file 1 — Supplementary Material 1. Figure S1. Images of 20 medicinal mollusk species from the Lake Taihu wetland. 1 (abc). Sinotaia quadrata; 2 (abc). Radix swinhoei; 3 (abc). Unio douglasiae; 4 (abc). Sinotaia purificata; 5 (abc). S. ningpoensis; 6 (abc). Limnoperna lacustris; 7 (abc). Pomacea canaliculata; 8 (abc). Radix plicatula; 9 (abc). Sinotaia aeruginosa; 10 (abc). Parafossarulus eximius; 11 (ab). Corbicula fluminea; 12 (abc). Corbicula nitens; 13 (ab). Polypylis hemisphaerula; 14 (ab). H. umbilicalis; 15 (ab). Gyraulus albus; 16 (ab). Hippeutis cantori; 17 (abc). Stenothyra glabra (S. glabra); 18 (abc). Parafossarulus striatulus; 19 (abc). Alocinma longicornis; 20 (ab). Gyraulus convexiusculus. Scale bar length: 18 mm [file 13071_2026_7273_MOESM1_ESM.jpg]

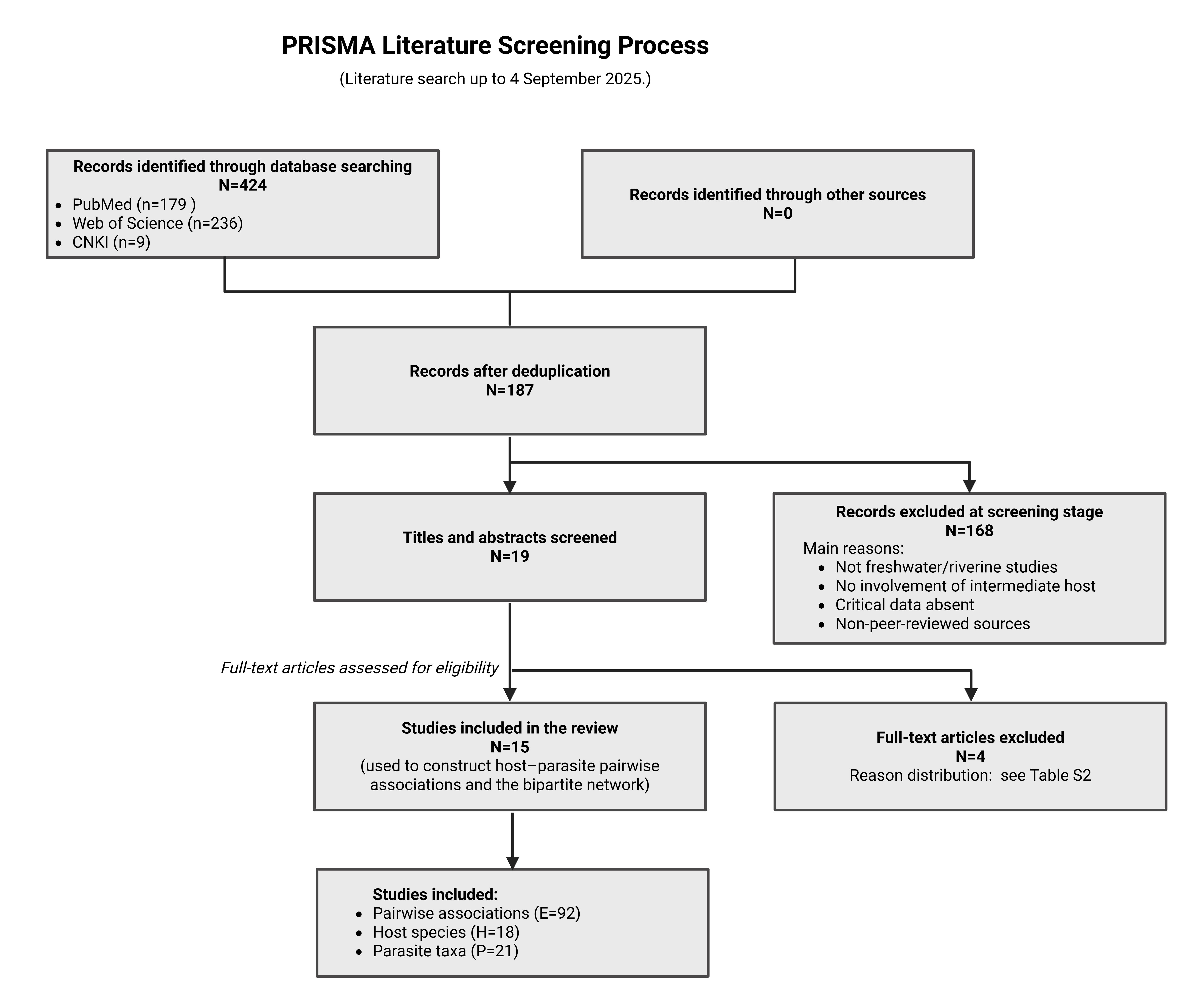

Supplement: Supplementary file 2 — Supplementary Material 2. Figure S2. Literature screening process for identifying key host-parasite associations. [file 13071_2026_7273_MOESM2_ESM.jpeg]
