# Supplementary material for: Ecological patterns and spatial distribution of medicinal mollusks in a freshwater ecosystem
Source: Parasit Vectors. 2026 Feb 18;19:119. doi: 10.1186/s13071-026-07273-9 (PMC12997700; doi:10.1186/s13071-026-07273-9)
Supplement: Supplementary file 3 — Supplementary Material 3. [file 13071_2026_7273_MOESM3_ESM.docx]

**Table S1. Topological parameters of each node in the mollusk–parasite interaction network.**

| **AverageShortestPathLength** | **BetweennessCentrality** | **ClosenessCentrality** | **ClusteringCoefficient** | **Degree** | **Eccentricity** | **IsSingleNode** | **name** | **NeighborhoodConnectivity** | **NumberOfDirectedEdges** | **NumberOfUndirectedEdges** | **PartnerOfMultiEdgedNodePairs** | **Radiality** | **selected** | **SelfLoops** | **shared name** | **Stress** | **TopologicalCoefficient** | **Type** |
| --- | --- | --- | --- | --- | --- | --- | --- | --- | --- | --- | --- | --- | --- | --- | --- | --- | --- | --- |
| 3.9 | 0 | 0.25641025641025644 | 0 | 1 | 7 | FALSE | Corbicula fluminea | 6 | 0 | 1 | 0 | 0.6777777777777777 | FALSE | 0 | Corbicula fluminea | 0 | 0 | Host |
| 5.275 | 0 | 0.1895734597156398 | 0 | 1 | 8 | FALSE | unknown | 2 | 0 | 1 | 0 | 0.5249999999999999 | FALSE | 0 | unknown | 0 | 0 | Parasite |
| 4.3 | 0.05 | 0.23255813953488372 | 0 | 2 | 7 | FALSE | Limnoperna lacustris | 1.5 | 0 | 2 | 0 | 0.6333333333333333 | FALSE | 0 | Limnoperna lacustris | 180 | 0.5 | Host |
| 4.3 | 0 | 0.23255813953488372 | 0 | 1 | 7 | FALSE | Sinotaia quadrata | 4 | 0 | 1 | 0 | 0.6333333333333333 | FALSE | 0 | Sinotaia quadrata | 0 | 0 | Host |
| 4.4 | 0 | 0.22727272727272727 | 0 | 1 | 7 | FALSE | Sinotaia aeruginosa | 2 | 0 | 1 | 0 | 0.6222222222222222 | FALSE | 0 | Sinotaia aeruginosa | 0 | 0 | Host |
| 5.025 | 0 | 0.1990049751243781 | 0 | 1 | 7 | FALSE | Parabucephalopsis prosorchis | 2 | 0 | 1 | 0 | 0.5527777777777777 | FALSE | 0 | Parabucephalopsis prosorchis | 0 | 0 | Parasite |
| 4.05 | 0.05 | 0.2469135802469136 | 0 | 2 | 6 | FALSE | Semisulcospira cancellata | 2 | 0 | 2 | 0 | 0.6611111111111111 | FALSE | 0 | Semisulcospira cancellata | 128 | 0.5 | Host |
| 3.375 | 0.09743589743589744 | 0.2962962962962963 | 0 | 2 | 6 | FALSE | Dollfustrema vaneyi | 5 | 0 | 2 | 0 | 0.7361111111111112 | FALSE | 0 | Dollfustrema vaneyi | 356 | 0.5 | Parasite |
| 3.425 | 0.05 | 0.29197080291970806 | 0 | 2 | 6 | FALSE | Echinochasmus japonicus | 4.5 | 0 | 2 | 0 | 0.7305555555555556 | FALSE | 0 | Echinochasmus japonicus | 180 | 0.5 | Parasite |
| 3.475 | 0 | 0.28776978417266186 | 0 | 1 | 6 | FALSE | Echinochasmus perfoliatus | 8 | 0 | 1 | 0 | 0.7250000000000001 | FALSE | 0 | Echinochasmus perfoliatus | 0 | 0 | Parasite |
| 3.475 | 0 | 0.28776978417266186 | 0 | 1 | 6 | FALSE | Metorchis taiwanensis | 8 | 0 | 1 | 0 | 0.7250000000000001 | FALSE | 0 | Metorchis taiwanensis | 0 | 0 | Parasite |
| 2.5 | 0.5234508547008545 | 0.4 | 0 | 8 | 5 | FALSE | Parafossarulus striatulus | 2.375 | 0 | 8 | 0 | 0.8333333333333334 | FALSE | 0 | Parafossarulus striatulus | 2044 | 0.1375 | Host |
| 3.425 | 0.05 | 0.29197080291970806 | 0 | 2 | 6 | FALSE | Asymphylodora japonica | 4.5 | 0 | 2 | 0 | 0.7305555555555556 | FALSE | 0 | Asymphylodora japonica | 180 | 0.5 | Parasite |
| 4.4 | 0 | 0.22727272727272727 | 0 | 1 | 7 | FALSE | Parafossarulus eximius | 2 | 0 | 1 | 0 | 0.6222222222222222 | FALSE | 0 | Parafossarulus eximius | 0 | 0 | Host |
| 3.125 | 0.12792200854700855 | 0.32 | 0 | 3 | 5 | FALSE | Paragonimus westermani | 4.666666666666667 | 0 | 3 | 0 | 0.7638888888888888 | FALSE | 0 | Paragonimus westermani | 442 | 0.36666666666666664 | Parasite |
| 4.525 | 0 | 0.22099447513812154 | 0 | 1 | 7 | FALSE | Metorchis orientalis | 4 | 0 | 1 | 0 | 0.6083333333333333 | FALSE | 0 | Metorchis orientalis | 0 | 0 | Parasite |
| 3.225 | 0.030486111111111103 | 0.31007751937984496 | 0 | 2 | 5 | FALSE | Haplorchis pumilio | 6 | 0 | 2 | 0 | 0.7527777777777778 | FALSE | 0 | Haplorchis pumilio | 190 | 0.5555555555555556 | Parasite |
| 3.55 | 0.09152777777777778 | 0.28169014084507044 | 0 | 4 | 6 | FALSE | Alocinma longicornis | 2 | 0 | 4 | 0 | 0.7166666666666667 | FALSE | 0 | Alocinma longicornis | 470 | 0.3333333333333333 | Host |
| 4.3 | 0 | 0.23255813953488372 | 0 | 1 | 7 | FALSE | Pomacea canaliculata | 4 | 0 | 1 | 0 | 0.6333333333333333 | FALSE | 0 | Pomacea canaliculata | 0 | 0 | Host |
| 3.975 | 0 | 0.25157232704402516 | 0 | 1 | 6 | FALSE | Haplorchis yokogawai | 4 | 0 | 1 | 0 | 0.6694444444444445 | FALSE | 0 | Haplorchis yokogawai | 0 | 0 | Parasite |
| 3 | 0.07169122544122544 | 0.3333333333333333 | 0 | 4 | 5 | FALSE | Hippeutis umbilicalis | 3.5 | 0 | 4 | 0 | 0.7777777777777778 | FALSE | 0 | Hippeutis umbilicalis | 386 | 0.4166666666666667 | Host |
| 2.95 | 0.033468822843822826 | 0.3389830508474576 | 0 | 4 | 5 | FALSE | Hippeutis cantori | 5 | 0 | 4 | 0 | 0.7833333333333333 | FALSE | 0 | Hippeutis cantori | 356 | 0.5714285714285714 | Host |
| 3.375 | 0.0638888888888889 | 0.2962962962962963 | 0 | 2 | 6 | FALSE | Haplorchis taichui | 5.5 | 0 | 2 | 0 | 0.7361111111111112 | FALSE | 0 | Haplorchis taichui | 404 | 0.5 | Parasite |
| 4.175 | 0 | 0.23952095808383234 | 0 | 1 | 8 | FALSE | Echinostoma cinetorchis | 7 | 0 | 1 | 0 | 0.6472222222222223 | FALSE | 0 | Echinostoma cinetorchis | 0 | 0 | Parasite |
| 3.2 | 0.14088157213157215 | 0.3125 | 0 | 7 | 7 | FALSE | Gyraulus convexiusculus | 3.2857142857142856 | 0 | 7 | 0 | 0.7555555555555555 | FALSE | 0 | Gyraulus convexiusculus | 1112 | 0.32653061224489793 | Host |
| 3.275 | 0.026792073667073663 | 0.3053435114503817 | 0 | 4 | 6 | FALSE | Echinostoma miyagawai | 5.25 | 0 | 4 | 0 | 0.7472222222222222 | FALSE | 0 | Echinostoma miyagawai | 292 | 0.4722222222222222 | Parasite |
| 2.75 | 0.11763588263588258 | 0.36363636363636365 | 0 | 6 | 5 | FALSE | Polypylis hemisphaerula | 4.5 | 0 | 6 | 0 | 0.8055555555555556 | FALSE | 0 | Polypylis hemisphaerula | 878 | 0.3888888888888889 | Host |
| 3.075 | 0.028589905464905464 | 0.3252032520325203 | 0 | 4 | 6 | FALSE | Echinostoma ilocanum | 6.5 | 0 | 4 | 0 | 0.7694444444444444 | FALSE | 0 | Echinostoma ilocanum | 350 | 0.4230769230769231 | Parasite |
| 3.125 | 0.020806647056647056 | 0.32 | 0 | 3 | 6 | FALSE | Fasciolopsis buski | 7.333333333333333 | 0 | 3 | 0 | 0.7638888888888888 | FALSE | 0 | Fasciolopsis buski | 254 | 0.48717948717948717 | Parasite |
| 3.675 | 0 | 0.272108843537415 | 0 | 1 | 6 | FALSE | Sanguinicola lungjiangensis | 9 | 0 | 1 | 0 | 0.7027777777777778 | FALSE | 0 | Sanguinicola lungjiangensis | 0 | 0 | Parasite |
| 3.675 | 0 | 0.272108843537415 | 0 | 1 | 6 | FALSE | Orientobilharzia turkestanicum | 9 | 0 | 1 | 0 | 0.7027777777777778 | FALSE | 0 | Orientobilharzia turkestanicum | 0 | 0 | Parasite |
| 2.7 | 0.19695792633292636 | 0.37037037037037035 | 0 | 9 | 5 | FALSE | Radix plicatula | 3 | 0 | 9 | 0 | 0.8111111111111111 | FALSE | 0 | Radix plicatula | 1114 | 0.2857142857142857 | Host |
| 3.625 | 0 | 0.27586206896551724 | 0 | 1 | 6 | FALSE | Trichobilharzia physella | 8 | 0 | 1 | 0 | 0.7083333333333334 | FALSE | 0 | Trichobilharzia physella | 0 | 0 | Parasite |
| 3.375 | 0.003717948717948718 | 0.2962962962962963 | 0 | 2 | 6 | FALSE | Trichobilharzia paoi | 8.5 | 0 | 2 | 0 | 0.7361111111111112 | FALSE | 0 | Trichobilharzia paoi | 44 | 0.6818181818181818 | Parasite |
| 3.325 | 0.10121184371184369 | 0.3007518796992481 | 0 | 4 | 6 | FALSE | Angiostrongylus cantonensis | 4 | 0 | 4 | 0 | 0.7416666666666667 | FALSE | 0 | Angiostrongylus cantonensis | 524 | 0.3 | Parasite |
| 3.125 | 0.030337301587301593 | 0.32 | 0 | 3 | 6 | FALSE | Euparyphium ilocanum | 6.333333333333333 | 0 | 3 | 0 | 0.7638888888888888 | FALSE | 0 | Euparyphium ilocanum | 276 | 0.41025641025641024 | Parasite |
| 2.325 | 0.45280108780108774 | 0.4301075268817204 | 0 | 6 | 4 | FALSE | Echinoparyphium recurvatum | 6.5 | 0 | 6 | 0 | 0.8527777777777777 | FALSE | 0 | Echinoparyphium recurvatum | 2164 | 0.275 | Parasite |
| 3.375 | 0.003717948717948718 | 0.2962962962962963 | 0 | 2 | 6 | FALSE | Echinostoma hortense | 8.5 | 0 | 2 | 0 | 0.7361111111111112 | FALSE | 0 | Echinostoma hortense | 44 | 0.6818181818181818 | Parasite |
| 2.925 | 0.10216413216413218 | 0.3418803418803419 | 0 | 6 | 6 | FALSE | Echinostoma revolutum | 5.833333333333333 | 0 | 6 | 0 | 0.7861111111111111 | FALSE | 0 | Echinostoma revolutum | 836 | 0.34523809523809523 | Parasite |
| 3.375 | 0.003717948717948718 | 0.2962962962962963 | 0 | 2 | 6 | FALSE | Fasciola hepatica | 8.5 | 0 | 2 | 0 | 0.7361111111111112 | FALSE | 0 | Fasciola hepatica | 44 | 0.6818181818181818 | Parasite |
| 2.65 | 0.20002696377696377 | 0.37735849056603776 | 0 | 8 | 5 | FALSE | Radix swinhoei | 3.25 | 0 | 8 | 0 | 0.8166666666666667 | FALSE | 0 | Radix swinhoei | 1070 | 0.25 | Host |

**Table S2. Mean and maximum densities (ind·m^-2^) of mollusk species in the northern Taihu Lake wetland.**

| **Species** | **Mean Density (ind·m^-2^)** | **Max Density (ind·m^-2^)** |
| --- | --- | --- |
| *R. swinhoei* | 0.6 | 16.0 |
| *R. plicatula* | 0.0 | 0.9 |
| *P. hemisphaerula* | 0.2 | 8.0 |
| *G. albus* | 0.0 | 0.9 |
| *G. convexiusculus* | 0.1 | 2.8 |
| *H. cantori* | 0.3 | 8.0 |
| *H. umbilicalis* | 0.8 | 35.2 |
| *P. canaliculata* | 1.1 | 16.7 |
| *A. longicornis* | 2.6 | 80.0 |
| *P. eximius* | 1.7 | 32.0 |
| *P. striatulus* | 4.7 | 40.0 |
| *S. ningpoensis* | 0.0 | 0.9 |
| *S. glabra* | 0.0 | 0.9 |
| *S. aeruginosa* | 58.0 | 1256.0 |
| *S. purificata* | 10.5 | 224.0 |
| *S. quadrata* | 1.1 | 17.6 |
| *L. lacustris* | 0.0 | 0.9 |
| *C. nitens* | 0.1 | 3.7 |
| *C. fluminea* | 0.1 | 3.7 |
| *U. douglasiae* | 0.0 | 0.9 |

Note: mean density represents the average density across all sampling sites, and max density indicates the maximum observed density at any individual site.
